# Recent Advances in MEMS-Based 3D Hemispherical Resonator Gyroscope (HRG)—A Sensor of Choice

**DOI:** 10.3390/mi13101676

**Published:** 2022-10-05

**Authors:** Ahmad Rahbar Ranji, Vijayakanthan Damodaran, Kevin Li, Zilang Chen, Shahpour Alirezaee, Mohammed Jalal Ahamed

**Affiliations:** 1 Department of Maritime Engineering, Amirkabir University of Technology, Tehran P.O. Box 15475-4413, Iran; 2 Department of Mechanical, Automotive and Material Engineering, University of Windsor, Windsor, ON N9B 3P4, Canada

**Keywords:** dynamic performance, fused silica, glassblowing, hemispherical resonator gyroscope, MEMS

## Abstract

Macro-scale, hemispherical-shaped resonating gyroscopes are used in high-precision motion and navigation applications. In these gyroscopes, a 3D wine-glass, hemispherical-shaped resonating structure is used as the main sensing element. Motivated by the success of macroscale hemispherical shape gyroscopes, many microscale hemispherical-shaped resonators have been produced due to the rapid advancement in semiconductor-based microfabrication technologies. The dynamic performance of hemispherical resonators depends on the degree of symmetry, uniformity of thickness, and surface smoothness, which, in turn, depend on the type of materials and fabrication methods. The main aim of this review paper is to summarize the materials, characterization and fabrication methods reported in the literature for the fabrication of microscale hemispherical resonator gyroscopes (µHRGs). The theory behind the development of HRGs is described and advancements in the fabrication of microscale HRGs through various semiconductor-based fabrication techniques are outlined. The integration of electrodes with the hemispherical structure for electrical transduction using other materials and fabrication methods is also presented. A comparison of different materials and methods of fabrication from the point of view of device characteristics and dynamic performance is discussed. This review can help researchers in their future research and engineers to select the materials and methods for µHRG development.

## 1. Introduction

Microelectromechanical systems (MEMS) technology enables the fabrication of miniature integrated systems that transform mechanical signals into electrical signals. The main part of MEMS is the transducer, which converts one form of signal/energy into another and includes both sensors and actuators. An actuator transforms energy to motion or converts electrical quantity to mechanical quantities [1] and a sensor in reverse converts mechanical quantity to electrical quantity [1,2]. The sensor can be mechanical, thermal, chemical/biological, radiant, magnetic or electrical. Mechanical sensors that typically sense strains, motion, forces and/or displacements are in the form of piezoresistive, piezoelectric, capacitive, and resonant sensors [3].

MEMS vibratory gyroscopes sense rotational motion. The majority are based on constant vibration of a resonating mass on a rotating body to induce the Coriolis force. Therefore, they are called Coriolis vibratory gyroscopes (CVGs). In CVGs, the resonating mass acts as the main sensing element and the geometry of it can be of different shapes. Based on the resonating mass, these types of gyroscopes can be in the form of a string [4], a single beam [5,6], U-shaped tuning forks (two parallel beams) [7,8,9], rocking-mass gyroscope systems (two perpendicular in-plane beams) [10,11], a butterfly [12,13], disks [14,15,16,17,18,19], cloverleaf-type disks [20], rings [21,22,23], rings with compliant spokes [24], non-continuous double rings (called folded-beam disks) [25], disks with special forms of supporting beams (called honeycomb disks) [26], cylinders [27,28,29,30,31], cupped cylinders [32], bell-shapes [33,34,35], 3D rectangular parallelepiped gyroscopes based on special vibratory modes of solid material (e.g., thickness-shear vibrating mode) [36,37], pierced shallow shells [38], shallow shells [39], microbubbles (spherical caps with a radius-over-height ratio larger than one) [40], cennospheres (hollow spheres) [41], quasi-spherical forms [42], spherical shell resonator gyroscopes [43,44,45,46], and, finally, hemispherical resonator gyros (HRGs).

HRGs are considered state-of-the-art, precise Coriolis vibratory gyros (CVGs), in which, in contrast to plane (flat) types, such as beams, rings, and disks, the unwanted parasitic out-of-plane modes of vibration are eliminated and there is high out-of-plane stiffness [47]. Accordingly, these gyroscopes are considered more accurate Coriolis vibratory gyroscopes [33]. HRGs have exhibited higher performance mainly because they are highly symmetric structures with low frequency mismatch, high insensitivity to environmental vibrations, an extended dynamic range, high precision, good impact resistance, and large bandwidth, together with long-lasting duration, making HRGs ideal sensors of choice for aircraft navigation, oil borehole exploration, planetary exploration and more [48,49]. Another important aspect is their low energy loss, meaning a higher Q-factor (quality factor). The Q-factor of a resonator is the ratio of stored energy to energy dissipated. A higher Q-factor means the resonator keeps oscillating longer, thus lowering loose of its energy. Other advantages of HRGs include an excellent quality factor, high-performance application [50], light weight, compactness, operation in a vacuum, negligible sensitivity to acceleration, and lack of moving parts [51].

Stimulated by the advantages outlined and the proven excellent performance of macroscale HRGs, in recent years much effort has been invested in finding novel ways to miniaturize HRGs to achieve wafer-scale manufacturability to minimize cost, size, weight and power (CSWaP) [52]. However, miniaturization of such 3D structures, while retaining symmetry and material properties, is challenging. The quality of the resonator can be impaired by material defects, residual stress, geometric parameter errors and other fabrication defects [53]. Building a gyroscope with a high-performance matrix at low cost is the most challenging task [50]. Overcoming these challenges has created many opportunities for researchers to explore the limits of fabrications and they have proposed various intriguing solutions. In recent years, many studies have reported different fabrication methods for HRGs and materials, which makes it necessary to produce an overview of these investigations. The main aim of this study is to review and summarize in-depth all the materials and fabrication methods that have been used to build different types of microscale HRGs, as well as their dynamics, resonance, damping and performance characteristics. In this review paper, we first provide an overview of HRG theory and performance in Section 2. In Section 3, we review state-of-the-art microscale HRG fabrication processes, materials and their performance. In Section 4, we present the conclusions and discuss possible future directions.

## 2. Theory and Performance of HRG

**Figure 1:**
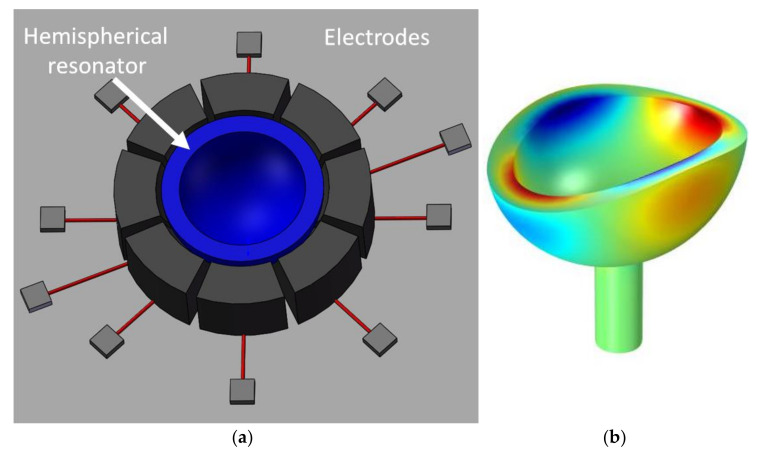
(**a**) Hemispherical shell (Liu et al. [55]), Reproduced with permission from ASME; published by J. Liu, J. Jaekel, D. Ramdani, N. Khan, D. S.-K. Ting and M. J. Ahamed, “Effect of Geometric and Material Properties on Thermoelastic Damping (TED) of 3D Hemispherical Inertial Resonator”, in Proceedings of the ASME 2016 International Mechanical Engineering Congress and Exposition, IMECE2016, Phoenix, AZ, USA, 11–17 November 2016, (**b**) Second mode of vibration (*n* = 2) of a wineglass (hemisphere)-shaped resonator (Ahamed et al. [56]) © 2022 IEEE. Reprinted, with permission from IEEE from M. J. Ahamed, D. Senkel and A. M. Shkel, “Effect of Annealing on Mechanical Quality Factor of Fused Quartz Hemispherical Resonator”, in 2014 International Symposium on Inertial Sensors and Systems (ISISS), Laguna Beach, CA, USA, 25–26 February 2014.

The performance of an HRG is based on accurate sensing of the first bending mode of vibration of an extremely symmetric hemispherical shell resonator. The circle deforms to an ellipse in the first quarter of the vibration cycle and returns to a circle in the second quarter. In the third and fourth cycles, it deforms to an ellipse and back to the circle, but the semimajor and semiminor axes are reversed [54]. A conceptual schematic of microHRG with surrounding electrodes is shown in Figure 1a [55] and *n* = 2 mode of vibration shown in Figure 1b [56]. 

These two symmetric and perpendicular bending deformations of the hemispherical shell are called the elliptical mode [53]. They are also referred to as a standing wave pattern since the position of the maximum displacements (lobes or antinodes) and minimum/null displacement (nodes) are stable with respect to the shell [51,57]. In the first mode of vibration (rocking mode), the hemisphere rotates as a rigid body around one axis. Thus, the vibration mode shown in Figure 1 is the second mode of vibration and is referenced by *n* = 2 in the literature. The *n* = 2 mode of vibration of the hemisphere has four equally spaced antinodes and four nodes in between (Figure 1) and is the lowest-order wineglass mode [48]. The operation of the gyroscope in the *n* = 2 mode of vibration is highly sensitive to the geometry of the shell [58].

**Figure 2:**
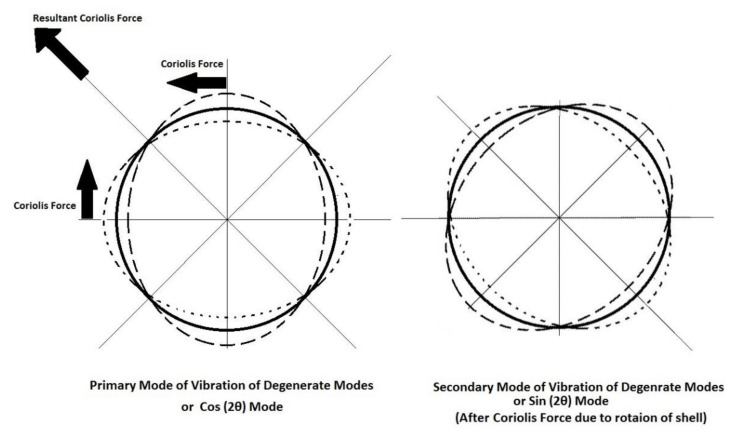
Degenerate modes of vibration of hemispherical shell in *n* = 2 mode.

For a fully symmetric shell without any restriction, there are an infinite number of elliptical axes of vibration in the *n* = 2 mode. A rotatory body generates the Coriolis force on an inside vibratory mass. In the *n* = 2 mode of vibration of an HRG, the Coriolis force is induced when the gyroscope rotates [58] (Figure 2). The induced Coriolis force is in the direction of a diagonal of 45° with respect to the axes of the ellipse, which creates another mode of vibration called the Coriolis measured mode or the sense mode [59], and the quadrature motion or the quadrature wave. Thus, there are a pair of elliptical vibration modes (Figure 2) called the two degenerate modes [54] or the drive and sense modes, and two cos(2*θ*) and sin(2*θ*) modes [59,60], which are coupled together through the Coriolis force [61]. Two modes of *n* = 2 have the same frequency if the resonator is highly symmetric, and a fraction of frequency mismatch leads to considerable bias stability. For example, a frequency split of *Δf* = 1 Hz results in a bias error of 360°/s [57]. The deviation of the sensor from its mean value of the output rate is called the bias stability (or bias instability), the zero-bias error [59], or the signal-to-noise ratio, which is a measure of the gyroscope stability over a specified period of time or quality of measurement [62]. Bias instability, which is a performance quality measurement of gyroscopes, is a drift of the measurement from the average value, also called the drift angle or bias drift, and has a unit of degree/h. The lower the bias stability is, the fewer the deviations from the mean rate over time. The angle random walk (ARW) is another performance quality measurement of a gyroscope and is defined as the average deviation or error when the signal is integrated and has the unit of degreeh.

**Figure 3:**
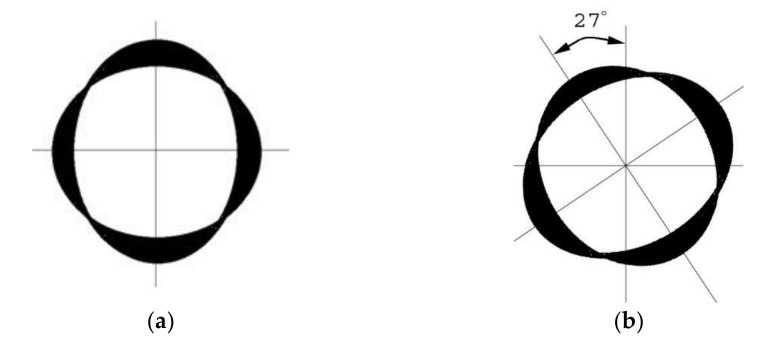
Rotation of standing wave due to the rotation of the gyro by 90° with respect to the stem. (**a**) Primary mode of vibration of HRG, (**b**) Secondary mode of vibration of HRG (the standing wave lag by 27°).

The energy transfers between these two degenerate modes by the Coriolis force cause the standing wave to lag behind the HRG by an angle in proportion to the angle of rotation of the HRG [63]. The ratio of the standing wave pattern to the physical rotation of the hemispherical shell is a precise value irrespective of the material properties. This ratio, which is approximately 0.3 [64], depends on the geometrical parameters of the shell and temperature [48], and is called the angular-gain factor, Ag, or the geometric scale factor. Figure 3 depicts a hemispherical shell in which the standing wave pattern lags by 27° when the shell is rotated by 90°. Dividing the angle of rotation of the standing wave with respect to HRG by the angular gain factor yields the angle of rotation of the shell. The precision of a gyroscope is due to the extreme stability of the gain factor [48].

**Figure 4:**
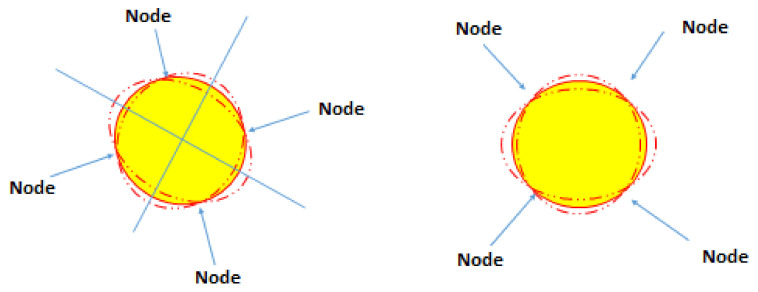
Two possible vibrating modes of an HRG (two standing wave patterns).

By applying an alternating electric (AC) voltage (actuator) in one of the two degenerate modes (the primary mode) of a gyroscope that is attached to a rotating body with angular rate, Ω the created Coriolis force excites the other mode (the secondary mode), through which Ω can be detected by electrical signals [58]. Figure 4 depicts two possible standing wave patterns out of an infinite number of possible wave patterns. The superposition of two in-phase wave patterns with the same and small amplitude produces another wave pattern. The most common standing wave pattern of HRGs fabricated with quartz is the superposition of two standing waves exactly 90° out of phase [63]. Control of an HRG involves setting the amplitude of the principal standing wave or antinodal wave to a desired value and nullifying the amplitude of the secondary standing wave or nodal quadrature [63].

Vibratory gyroscopes measure either the rotation rate (Ω) or angle (*θ*). When the angle is measured, it is operated in whole angle (WA) or rate-integrating, gyroscope (RIG) mode. If the rotation rate is measured, it is operated in force rebalance (FR), rate gyroscope (RG), or amplitude modulator (AM) mode [65]. The stability of the scale factor and the wide bandwidth and range of measurement, together with direct measurement of angle, are advantages of a RIG [66,67]. Rate gyroscopes can operate in open-loop or closed-loop modes. In open-loop operation, the sense mode is free to vibrate, and its amplitude is detected, since the ratio of the sense amplitude to the drive amplitude is proportional to the rotation rate of the shell. In closed-loop operation, an exerted force suppresses the sense mode, which is why it is called the force rebalance mode, and the amount of exerted force is detected, since the ratio of the exerted force to the drive force is proportional to the rotation rate of the shell. During the rotation of the HRG, the standing waves precess freely in WA mode while being fixed at a position in FR mode. The readout system in FR mode is much simpler than in WA mode [68]; however, the geometry needs to be absolutely symmetric and to have a very long decay time constant. Operating in WA mode leads to good scale factors and is preferred for a larger range of applications [64,69].

Electrodes are used to force the HRG to vibrate and to detect the flexural mode. Electrostatic types of electrode for driving (excitation) and capacitive electrodes for sensing (detection) are the most general form of electrodes used in Coriolis vibratory gyroscopes (CVGs). Angular vibration, line vibration and standing wave vibration are primary forms of electrostatic excitation, while angular vibration and line vibration are the main capacitive detection structural forms [65]. Thus, various combinations of drive-sense mode can be used. A hemispheric shell resonator is excited by externally and circumferentially placed electrodes around the hemispherical shell using standing wave vibrations. Signals from the HRG, with information about the amplitude and location of the standing wave on the shell, may be capacitively obtained [63]. The method of controlling this field determines the mode of operation of an HRG. The drive/excitation (actuation/forcer) electrodes are used to keep the standing-wave amplitude constant and to nullify the quadrature standing wave (secondary standing wave) amplitude (WA/RIG mode) or to keep the standing wave at a specific case-fixed location (FR/RG mode) [48].

The second set of electrodes are placed internally and circumferentially around and very close to the shell to sense the position and amplitudes of the antinodes and nodes. The displacement of the hemisphere shell is measured by pickoff electrodes with changes in the capacitance due to oscillation of the shell [64]. They also measure the location of the standing wave and the resonant behavior of the shell since the capacitance of each electrode is adjusted at the resonator bending frequency [48]. The error in determining the position of the standing wave and its movement, even in the absence of the inertial rate, makes it impossible to accurately estimate the input inertial rate [48]. The errors can be reduced using tuning electrodes, which diminish the mismatch frequency of two degenerate modes.

Pai et al. [70] discussed three different configurations of electrodes: electrostatic driving/sensing, piezoelectric-enhanced electrostatic driving/electrostatic sensing and electrostatic driving/optical sensing. Senkal et al. [71,72] discussed two sets of excitation and detection modes, namely, electrostatic excitation and detection and mechanical pinging and optical pick-up [73]. Cho et al. [74] compared different electrode integration approaches for HRGs and proposed a method for forming uniform electrodes around an HRG.

Two types of Coriolis vibratory gyroscopes (CVGs) are classified based on mechanical elements [75], i.e., degenerate and nondegenerate mode gyroscopes. Most of the MEMS CVGs are designed to operate as non-degenerate mode devices, although degenerate mode operation has the potential advantages of high rate sensitivity and whole angle operation [75].

Any resonating structure is subject to several energy losses, such as thermoelectric dissipation (TED), viscous damping, anchor losses, material losses, surface losses, and fluid losses. Loss of energy depends on the device geometry, material, environment, and operating frequency range. Fluid losses are insignificant if the HRG is working in a vacuum [60]. HRG has the lowest anchor loss, since the anchor is located at the stem (bottom), which is the position of a vibrational node and, thus, causes little interference with the vibration of the shell [57,63]. TED is the most important mechanism of energy dissipation in MEMS due to coupling of thermal perturbation and mechanical vibration [60]. Resonator shells with constant radius along the z-axis have lower anchor loss [76].

The Q-factor (quality factor) is a commonly used parameter that accounts for energy dissipation and is defined as the ratio of the total energy of the resonator to the energy lost per cycle, which is inversely proportionate to damping. Systems with a high Q-factor have a large and sharp response with low energy loss when driven at resonant frequencies [77]. The selection of an HRG is based on the Q-factor of the resonator. Using an electrostatic system for excitation and detection of the vibration of a shell resonator, using the inertial properties of a standing wave, results in extremely low specific energy losses [78].

Small errors in the resonator create damping and degrade its performance and must be controlled by signal processing and feedback control loops [63]. Two control loops are required for the FR mode. One is to force the resonator to vibrate at its natural frequency (resonance), while the second is to nullify the displacements at the nodes [49].

## 3. Microscale HRG Fabrication Methods and Materials

The ability to shrink macroscale hemispherical shells is the main reason for progress in *μ*HRG. Although reducing the size of an HRG has many advantages, such as low cost and small power dissipation, it increases the frequency mismatch [79]. In general, the smaller the size of an HRG, the more noise, less sensitivity (scale factor) and driving force there is [51].

HRGs are built in either wineglass or domed configurations [54]. Two essential requirements for HRG operation are low resonance frequency mismatch, Δf between the two degenerate resonance modes (symmetric shell), and long resonance decay time, τ=Qπf0 (low resonance frequencies, $f_{0}$, and high-quality factors, *Q*) [80]. A high-quality factor imposes challenging requirements for resonator materials (i.e., a low coefficient of thermal expansion and high thermal conductivity) and dimensional control (uniform thickness) [54]. Wineglass resonators are applicable over a wide range due to their inherently high Q-factor [81]. Minimizing the dimensions of MEMS resonators increases the resonant frequency. However, the ratio of surface to volume also increases, leading to a decrease in the quality factor due to increased surface losses.

Microfabrication methods of MEMS were limited to planar processes (in the plane or perpendicular to wafer surface) and were not applicable to batch fabrication of 3D shell microstructures due to low accuracy [54]. Advanced applications of MEMS require sophisticated 3D shells, which has motivated new research into fabrication processes. Silicon as a MEMS material can achieve a quality factor of over a million, leading to a large Coriolis force [82]. MEMS wineglass structures are made using two different methods: deposition of thin films on predefined molds and blowing the device layer into predefined cavities [83].

### 3.1. The First Microscale HRG Demonstration: The Birth of the Glassblowing Technique

To the best of our knowledge, Eklund and Shkel [84,85] were the first to report the microscale fabrication of a shell using glassblowing techniques at a wafer level. Fabrication starts by patterning a silicon wafer with a layer of photoresist. Timed deep reactive-ion etching (DRIE) is applied to etch cylindrical cavities in the silicon wafer. The silicon wafer is bonded with a thin borosilicate glass wafer (Pyrex 7740) covering the etched cavities. The bonded wafers are sent to the furnace and heated at atmospheric pressure with a temperature above the softening point of glass (approx. 850 deg.). The glass deforms into 3D spherical shapes due to the inside cavity air pressure. The heated wafers are slowly cooled at room temperature. To remove residual stresses at the end of the fabrication processes, an annealing step may be applied.

Shaping glass is possible due to the high dependency of viscosity on temperature. Glass-blowing techniques are used to shape glass on the microscale by heating above the softening point. First, a glass wafer is bonded to a through-etched silicon wafer, followed by attachment of a blow hose to blow spheres when the glass is heated. Achieving a good seal between the silicon wafer and the blow hose to withstand high temperatures is a challenging task. The increased pressure of the small air pockets trapped between the glass and silicon during anodic bonding forces the glass to deform in unexpected areas of the wafer glass. A new approach to this process involves etching deep cavities in silicon to increase the volume of trapped air, similar to the process of plastically deforming silicon [84]. This technique, in combination with integrated electrodes, is promising for the fabrication of HRGs [86].

### 3.2. Use of Fused Silica (FS) in the Glassblowing Process to Fabricate a Microhemispheric Shell

While the first micro-shell fabrication was demonstrated using borosilicate glass (Pyrex), the impurity in borosilicate and its thermal coefficient compared to quartz limit its ability to attain higher Q-factor. Fused silica is preferred to borosilicate glass due to its low thermal coefficient and low internal thermo-elastic dissipation. The heat flow due to the coupling of mechanical vibration and thermal alteration of materials, such as silicon, leads to high TED. Using fused quartz and ultralow expansion titania silicate glass (ULE TSG) with low heat transfer is a potential solution [84]. However, the softening point of fused silica is much higher at upwards of 1600 degrees which makes it challenging to apply in micro-scale glassblowing. For the first time, Senkal et al. [87,88] used fused quartz and ULE TSG for the fabrication of MEMS micro-shell structures using an in-house glassblowing process capable of glassblowing at 1800 deg. with rapid 500 deg./min cooling.

**Figure 5:**
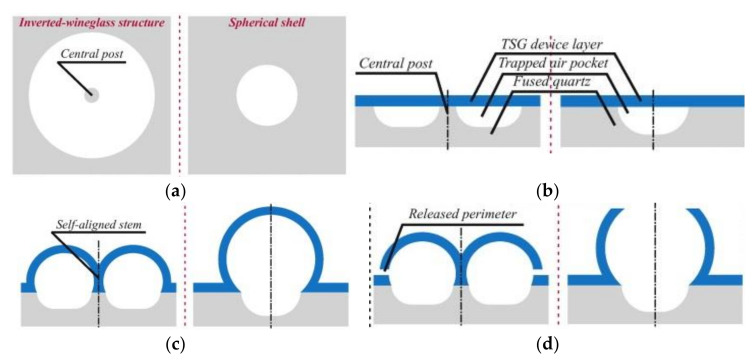
Schematic showing step-by-step process for ULE TSG/fused-quartz-based micro-glassblowing process (Senkal et al. [87]). Reprinted from [87], © (2022), with permission from Elsevier. (**a**) Footprint of the desired geometry is patterned on FS, (**b**) FS is etched and TSG device layer is bonded on top, (**c**) TSG/FS stack is glass blown, (**d**) Wineglass structure is released along the perimeter.

**Figure 6:**
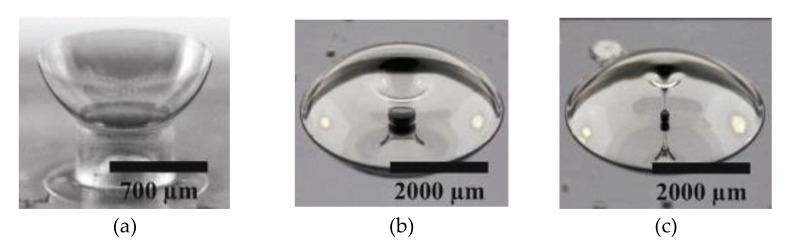
Pictures showing ULE TSG/fused-quartz final fabricated 3D wineglass and spherical shells (Senkal et al. [87]). Reprinted from [87], © (2022), with permission from Elsevier. (**a**) Hemisphere with hollow stem, (**b**) Hemisphere with solid stem of diameter 500 μm, (**c**) Hemisphere with solid stem of diameter 60 μm.

Apart from glassblowing at a much higher temperature, a further challenge is to be able to etch deep cavities, stem, and gaps on fused silica. Fabrication processes (Figure 5 [87]) start with deposition of a hard etch mask onto the fused quartz substrate wafer. Holes are introduced on the mask using reactive-ion etching (RIE). Wet etching of fused silica using concentrated HF (hydrofluoric acid) is used to etch deep cavities and stems into the bottom substrate. The etch mask layer is then stripped off and cleaned thoroughly using an RCA cleaning process. Plasma activation is applied to bond the top device layer onto the etched bottom substrate layer. TSG/fused-quartz-bonded wafers were then glass blown in the furnace at a temperature of approximately 1650 deg. and rapidly cooled. When heated, the air trapped in the bottom cavity expands while the top device layer becomes viscous at around its softening point. Because the two layers are bonded, the pressure in the trapped air increases with increase in temperature making the viscous top layer deform and create a hemispherical shape. Finally, laser ablation is used to release the shell, as shown in Figure 6 [87,88,89].

Advantages of this process are invariant material properties, no recrystallization and cracking, high atomic smoothness of the surface of TSG, extremely symmetric structures, and resultant high Q-factor resonators. Figure 6 shows the final fabricated hemispheres with hollow and solid stems.

### 3.3. Wafer-Scale Shells Fabricated by Glassblowing with Integrated Electrode Structures (Microscale HRGs)

**Figure 7:**
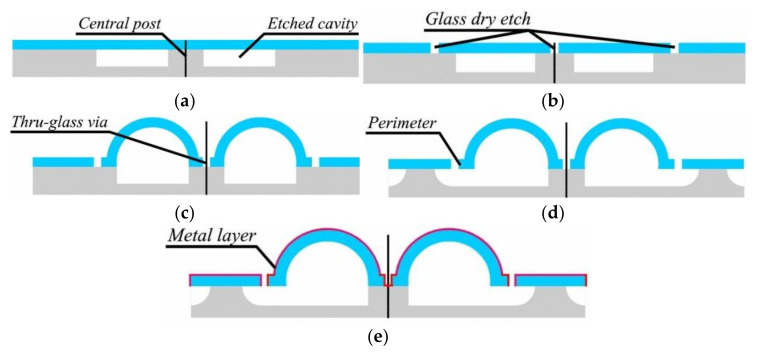
Step-by-step process flow diagram showing micro-glassblowing-based fabrication process of wineglass resonator with in-plane electrodes (Senkel et al. [86,90]) © 2022 IEEE. Reprinted, with permission from IEEE from D. Senkal, M. J. Ahamed, A. A. Trusov and A. M. Shkel, “Demonstration of sub-1 Hz structural symmetry in micro-glassblown wineglass resonators with integrated electrodes”, in Transducers & Eurosensors XXVII: The 17th International Conference on Solid-State Sensors, Actuators and Microsystems (TRANSDUCERS & EUROSENSORS XXVII), Barcelona, Spain, 16–20 June 2013. (**a**) Etching Silicon substrate and bonding glass layer, (**b**) Etching glass layer, (**c**) Glassblowing and forming 3-D shell, (**d**) Etching to release wineglass, (**e**) Coating outside of shell.

Apart from achieving micro-hemispherical shell structures with glassblowing at high temperatures, on-chip transduction and sensing by co-fabrication or integration of electrodes is a challenge. Adding an electrode for electrostatic transduction in wafer-scale 3D shells fabricated by the micro-glassblowing process enables this technique to be applied to build HRGs [73]. Senkel et al. [86,90] used micro-glassblowing to fabricate symmetric wineglass resonators from borosilicate glass with self-aligned stem structures and integrated electrode structures (Figure 7). Using DRIE (deep reactive-ion etching), cylindrical cavities are etched with a central post on a silicon substrate wafer, followed by anodic bonding of a thin glass layer onto the silicon substrate. The outer perimeter of the wineglass resonator and a central hole are defined using deep glass dry etching. Deep etching of glass is challenging requiring optimization of etch mask and dry etch parameters [91]. Deep trenches are etched using a thick, low-stress electroplated Cr/Ni hard etch mask. After removal of the etch mask, the wafer stack is micro-glass blown inside an RTA (rapid thermal annealing) system to create the 3D shell structure, followed by rapid cooling. The resonator is released by XeF_2_ (xenon difluoride) etching from the substrate underneath the glass layer along its perimeter and then blanket metallization by sputtering [86,90].

**Figure 8:**
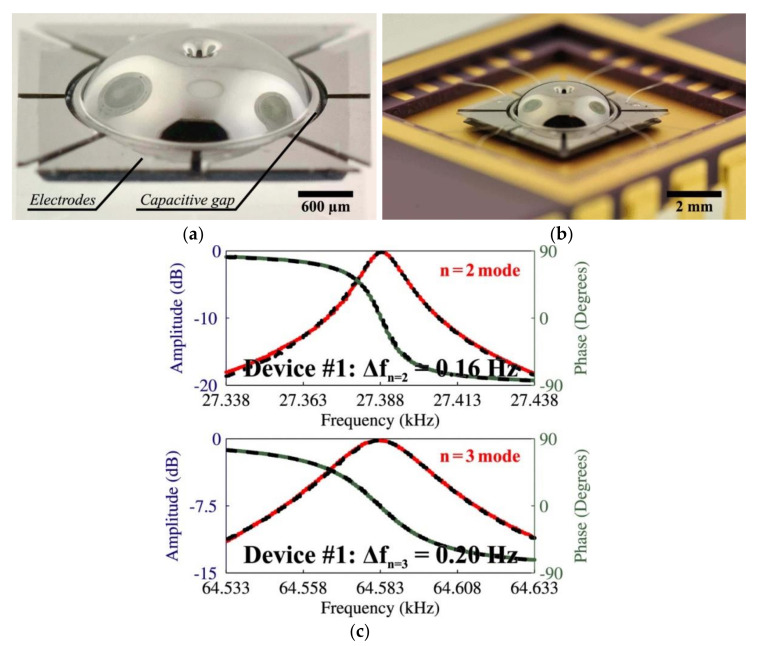
Metallized micro-wineglass structure with integrated electrodes, diameter 4 mm, thickness 50 µm, and its performance (Senkel et al. [90]) © 2022 IEEE. Reprinted, with permission from IEEE from D. Senkal, M. J. Ahamed, A. A. Trusov and A. M. Shkel, “Achieving sub-Hz frequency symmetry in micro-glassblown wineglass resonators”, Journal of Microelectromechanical Systems, vol. 23, no. 1, pp. 30–38, 2014. (**a**) Picture shwoing metallized micro-wineglass structure with integrated electrodes, (**b**) Picture showing packaged and wirebonded device, (**c**) Experimental frequency sweeps of *n* = 2 and *n* = 3 wineglass modes.

Testing of HRGs revealed that the *n* = 2 and *n* = 3 wineglass modes of vibration had a center frequency of 27,389 Hz in one prototype, with a sub-Hz frequency split, achieving highly symmetric structures [90]. Figure 8 shows the final fabricated hemispheres with integrated electrodes and frequency response showing a sub-Hz frequency split of the resonator [90].

The advantages of this fabrication method are its high structural symmetry because of the uniform temperature and gas flow. Edge defects and thermal/mechanical perturbations are eliminated during glassblowing and the wafer alignment steps, which are the main source of the frequency mismatch. Combination of the two lithography steps and use of a self-aligned stem reduce the mask misalignment. Elimination of etch asymmetry is achieved using anisotropic dry-etching. Using integrated electrode structures within the glass device layer leads to smoothness of surfaces. Thermal stress is lowered using the same materials for the resonator and electrode and uniform heating and cooling of wafers. A lower Q-factor and larger capacitive gaps represent challenges when using this method of fabrication.

**Figure 9:**
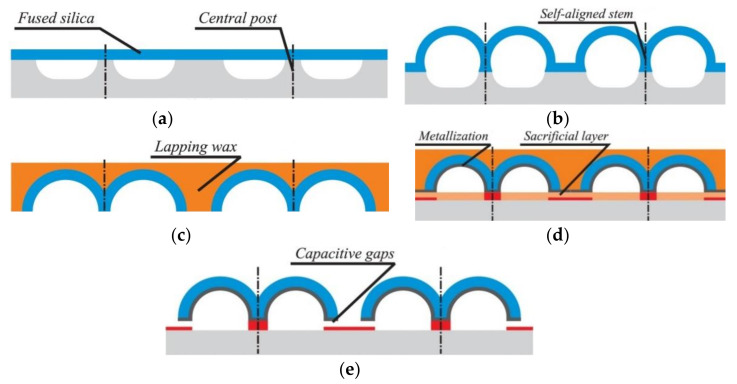
Step-by-step fabrication process of fused silica wineglass resonator using micro-glassblowing (Senkal et al. [75]) © 2022 IEEE. Reprinted, with permission from IEEE from D. Senkal, M. J. Ahamed, M. H. Asadian Ardakani, S. Askari and A. M. Shkel, “Demonstration of 1 million Q-factor on microglassblown wineglass resonators with out-of-plane electrostatic transduction”, Journal of Microelectromechanical Systems, vol. 24, no. 1, pp. 29–37, 2014. (**a**) Bonding wafer layer to pre-etched substrate, (**b**) Microglass blowing, (**c**) Removal of substrate by back-lapping, (**d**) Bonding the wineglass wafer to electrode wafer, (**e**) Removal of sacrificial layer from capacitive gaps.

**Figure 10:**
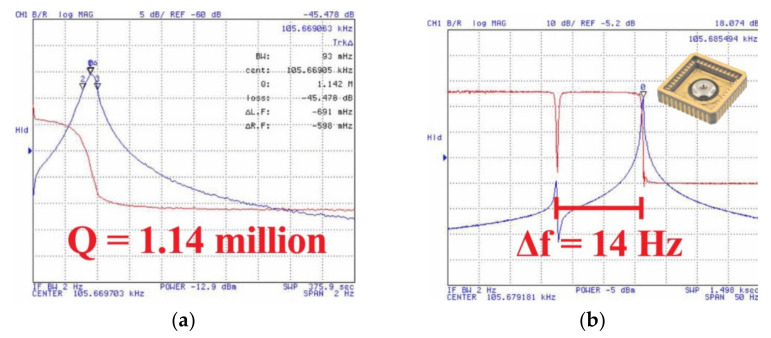
Performance of the HRG (Senkal et al. [75]) © 2022 IEEE. Reprinted, with permission from IEEE from D. Senkal, M. J. Ahamed, M. H. Asadian Ardakani, S. Askari and A. M. Shkel, “Demonstration of 1 million Q-factor on microglassblown wineglass resonators with out-of-plane electrostatic transduction”, Journal of Microelectromechanical Systems, vol. 24, no. 1, pp. 29–37, 2014. (**a**) Q-factor and (**b**) Frequency response.

To achieve a higher Q-factor and improve the sensing and actuation method, Senkal et al. [75,83,92,93] used a similar micro-glassblowing technique on fused silica with out-of-plane electrodes. Using this method, they achieved a Q-factor of one million and highly symmetric (Δff=132 ppm) HRG [75,83,92,93]. FS was used as the device material and for out-of-plane electrostatic transduction. A total of eight electrodes were used, which was the minimal configuration to drive and sense the *n* = 2 wineglass modes; four electrodes were designated forcers, and four were designated for pickoffs [89]. The fabrication process (Figure 9 [75]) started with LPCVD polysilicon etch-mask deposition on thick FS wafers. The polysilicon layer acted as an etch mask and was later patterned lithographically and used to etch cavities into the bottom FS wafers. Then, the polysilicon etch-mask was removed, and an FS device layer was bonded using plasma-assisted fusion bonding. The bonded wafer was then placed inside a furnace for glassblowing. After glassblowing, it was cooled rapidly to room temperature. The wafer stack was back-lapped to release the shells. The inside surface of the shells was metallized with sputtered iridium. FS out-of-plane electrode structures were fabricated on a separate wafer. Subsequently, the wineglass device was bonded to the electrode wafer at the stem of each wineglass. Then, the inverted wineglass was released by removing the sacrificial layer around their perimeter. The performance of the micro-HRG was measured experimentally, showing a Q-factor of 1.14 million and a frequency split of 14 Hz, as shown in Figure 10, at a center frequency of 105 kHz (Δff=32 ppm) and with a decay time of 3.18 s [92].

**Figure 11:**
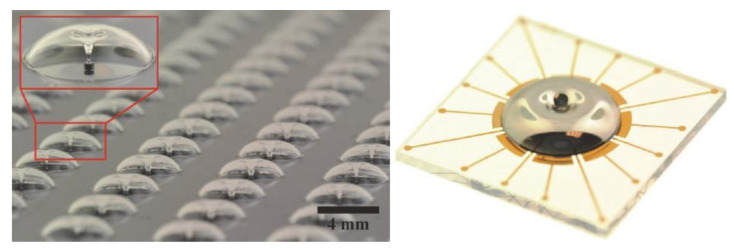
Image showing wafer-scale glassblowing can enable true batch micro-glassblowing fabrication process and final fabricated FS resonator with out-of-plane electrodes (Senkal et al. [92]) Reprinted from ref [92], © (2022), with permission from Elsevier.

Advantages of this method of fabrication are a high Q-factor of over one million for both *n* = 2 wineglass modes, a self-aligned glassblowing stem structure, and increased symmetry of frequency (Δff). Surface roughness and structural imperfections were minimized, leading to high smoothness and structural symmetry, which is impossible to achieve using conventional fabrication methods. The capacitive gap was uniform due to the addition of sacrificial layers and the elimination of misalignment by employing wafer-to-wafer bonding techniques. The same material was used for the electrode and the resonator, leading to the same thermal expansion. Disadvantages of the process included a larger frequency split, potentially large capacitive gaps, a small polar plate area and low transduction efficiency [94]. Figure 11 shows the final fabricated hemispheres with integrated electrodes. Studies have demonstrated that further improvement of the Q-factor can be achieved using a post-fabrication annealing process with a fused silica device [56].

**Figure 12:**
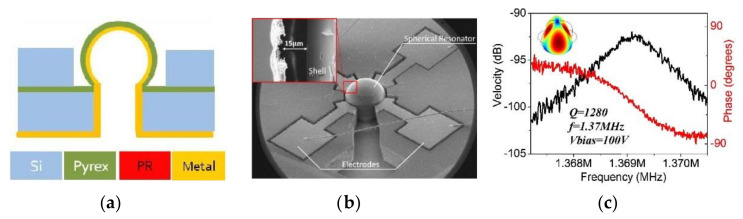
(**a**) Schematic showing design and different layers of final stage of coated Pyrex HRG with ALD coating, (**b**) and fabricated HRG, (**c**) Frequency and phase response of HRG in *n* = 2 mode of vibration (Giner et al. [95]) © 2022 IEEE. Reprinted, with permission from IEEE from J. Giner, J. M. Gray, J. Gertsch, V. M. Bright and A. M. Shkel, “Design, fabrication, and characterization of a micromachined glass-blown spherical resonator with in-situ integrated silicon electrodes and ALD Tungsten interior coating”, in 2015 28th IEEE International Conference on Micro-Electromechanical Systems (MEMS), Estoril, Portugal, 18–22 January 2015.

Uniform and high quality metalization of the 3D shell can be achieved using the ALD (atomic layer deposition) technique to obtain a full conformal and thin metal layer. Giner et al. [95] employed a glassblowing method to fabricate HRG using Pyrex as a wafer material, and, in the inner part of the shell, an ALD metal coating was applied. Figure 12 depicts a schematic and picture of the final product and performance of the resonator, respectively.

Advantages of this method of fabrication are the integration of electrodes as a part of micro-glassblowing, which reduces the complexity of the fabrication process by eliminating the extra fabrication step of electrodes. Additionally, there is the possibility of reducing the electrostatic gap since the gap width is not limited by the lithographic effect.

Asadian and Shkel [96] and Asadian et al. [47,97] used a high-temperature single glassblowing technique to fabricate a 3D fused-quartz (FQ) resonator in the form of a dual shell architecture with a common inner stem. The advantages of this anchoring were improvement of the structural rigidity, high-g shock survivability, and elimination of the risk of initiation of microcracks. An open-loop RIG transduced with electrostatic excitation and capacitive detection electrodes exhibited a drift angle of 0.4 deg.h [97].

**Figure 13:**
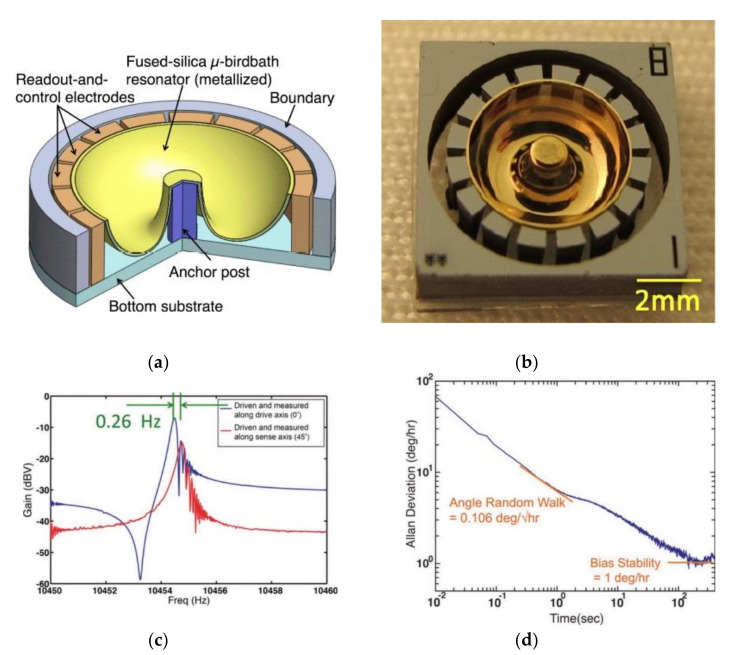
(**a**) Architecture of birdbath gyroscope (Woo et al. [104]), (**b**) final fabrication of BRG (Woo et al. [104]), © 2022 IEEE. Reprinted, with permission from IEEE from J. -K. Woo, J. Y. Cho, C. Boyd and K. NajafI, “Whole-angle-mode micromachined fused-silica birdbath resonator gyroscope (WA-BRG)”, in MEMS 2014, San Francisco, CA, USA, January 2014 (**c**) Frequency response (Cho et al. [69]), (**d**) Allan deviation plot (Cho et al. [69]) © 2022 IEEE. Reprinted, with permission from IEEE from J. Y. Cho, J. K. Woo, R. L. Peterson and K. Najafi, “Fused-Silica Micro Birdbath Resonator Gyroscope (μ-BRG)”, Journal of Microelectromechanical Systems, vol. 23, no. 1, pp. 66–77, February 2014.

In contrast to micro-scale high-temperature glassblowing inside a furnace, another process based on thermos-forming of fused silica involves using a blow-torch technique. Cho et al. [50,69,82,98,99,100], Shiari et al. [101], Nagourney et al. [102,103], Woo et al. [104], and Singh et al. [52] fabricated a microscale shell gyroscope using a blow-torching process assembled on an electrode substrate called the birdbath resonator gyroscope (BRG) (Figure 13). This is another type of wineglass design where the stem is located outside of the hemisphere.

Fabrication of molded substrates starts by micromachining the top and bottom molds. At the bottom of the cavity, through-holes are made to control the pressure across the substrate in the later steps. While the pressure inside the cavity decreases, the substrate, which is fixed between the top and bottom molds, is heated above its softening temperature using blow-torching. The pressure difference across the molding material and the bottom mold pulls the substrate down into the bottom cavity [98,105].

Assembly of the electrode substrate and resonator starts by deep-etching trenches on a P-type Si wafer using deep reactive-ion etching (DRIE). The top, sidewall, and bottom surfaces of the trenches are protected with an evaporated aluminum layer, which is then patterned by wet-etching. The Si wafer is bonded to a borosilicate [100], Pyrex [69], or FS [82,105] wafer. After removing Si from the topside using DRIE, Si electrodes remain with an anchor post. Dilute hydrochloric acid is used to etch the Al protection layer, leading to the release of Si pieces located between the anchor post and the electrodes. Polymer adhesives attach the resonator to the anchor post [69,100].

The *n* = 2 wineglass mode of vibration is measured and controlled by 16 electrodes located at the outer perimeter. Testing the BRG at <1 mTorr and room temperature revealed that the frequencies of the *n* = 2 wineglass modes were 10,465 and 10,479 Hz, with a frequency mismatch of $\Delta f_{n=2}=$ 14 Hz in forced-rebalance (FR) mode with a scale factor of 27.9 mV/(deg./s), and a bias stability of 1 deg.h. This split was reduced to 0.26 Hz after electrical tuning. The decay time constants were 7.45 s ($Q_{n=2}=244.75\;k$) and 7.69 s ($Q_{n=2}=252.64\;k$). The result of finite element analysis (FEA) gave 0.25, while the measurement showed 0.317 [69]. The performance of the HRG further improved with the quality factor raised to 5.2 million and the bias stability reduced to 0.0014 deg.h.

The advantages of this fabrication method are axisymmetric geometry and the possibility of fabricating with improved symmetry, reduction in anchor loss, low vibration sensitivity, and the possibility of trimming of *f* and Q by adding or removing mass on the rim of the resonator. Using FS as a device-layer material, in addition to having high fracture toughness, leads to less energy loss at the grains [82]. Disadvantages are a nonuniform capacitive gap, low transduction efficiency, and a complex fabrication process [94].

Singh et al. [106] used the same birdbath shell geometry to investigate the possibility of using the *n* = 3 wineglass mode of vibration for sensing. Darvishian et al. [107] numerically and analytically studied the TED in BRGs. Cho et al. [74] used the same fabrication method to build HRGs with reverse geometry.

The size and position of the anchor (stem) of the HRG has a great influence on the Q-factor [108]. Cho and Najafi [109] used the same method of fabrication process discussed in [82,85,98,99,100,101,102] to build an HRG with a solid stem, which had the advantages of low anchor loss due to the large stem length/stiffness ratio and small shell rim thickness. At <10 μTorr vacuum, the *n* = 2 wineglass mode had a frequency of 22.6 kHz and Q = 2.55 million [109].

Li et al. [110] used a combination of a blow-torching process and a highly precise mold to fabricate FS HRGs with the same method as [69,82,98,99,100,101,102,103,104]. Using a high-precision machining process, upper and lower graphite molds were fabricated.

**Figure 14:**
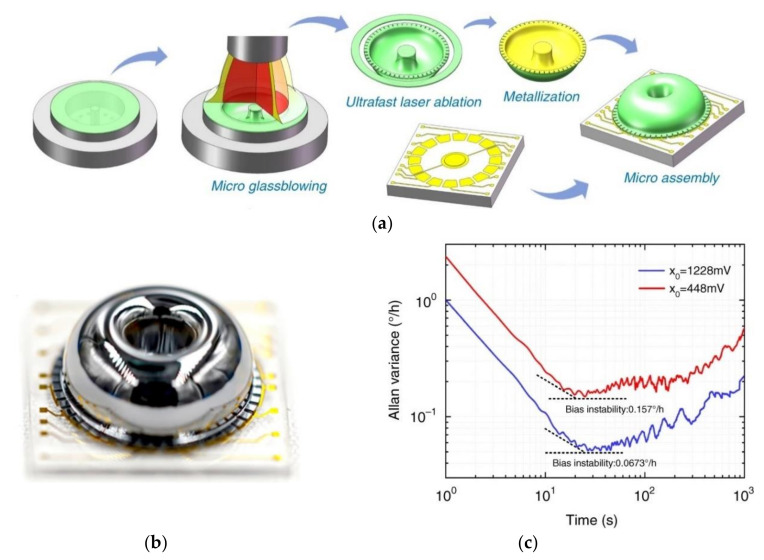
(**a**) Designs showing step-by-step fabrication process, (**b**) final product, (**c**) Allan deviation plot of the HRG developed by Sun et al. [120]. Reprinted from [120] under the terms of the Creative Commons CC BY license.

Xiao et al. [94], Lu et al. [111,112,113], Li et al. [114,115,116], and Shi et al. [117,118,119] improved the fabrication method of Li et al. [110] by putting molds on an adjustable whirling platform and using a femtosecond ablation method to release the shell with T-shape masses to enhance the symmetry of the shell resonators and decrease roughness. Figure 14 shows the fabrication process, final product and performance of the HRG (Sun et al. [120]). Advantages of this method of fabrication are high radial symmetry and surface quality, increased area of the electrode, small and uniform capacitive gaps, and the possibility of changing the distribution of the effective masses and stiffness of the shell to trim the frequency by adjustable T-shape masses. Sun et al. [121] studied the source and effect of phase errors, and Sun et al. [122] investigated the angle drift due to errors in the actuation electrode of this HRG when worked in WA mode. The quality factor of the HRG was increased and the drift angle was reduced significantly to 0.0673 deg.h [120].

Wang et al. [123,124] used Pyrex 7740 glass (borosilicate glass) to fabricate HRG by the glassblowing method. Advantages of this method of fabrication were improved metal malleability, elimination of metal cracking during glass expansion, reduction in applied voltage, and increased capacitance.

### 3.4. Other Materials and Fabrication Methods

Apart from borosilicate and fused silica, other materials have also been investigated to produce 3D hemispherical shells, with some approaches using thermoforming of other materials, while others used deposition/release. Sarac et al. [81] introduced bulk metallic glasses (BMGs) to build HRGs using a blow-molding method. The BMG was heated to the processing temperature, and a pressure gradient was forced to fill a cavity. Then, it was bonded to the Si wafer by a mechanical process. A thin layer of photoresist was spun onto the silicon wafer and then patterned using basic photolithography. Using DRIE, the exposed areas of Si were etched in such a way that the depth of etching of large holes was all the way through the Si and the small holes in relation to their diameter. The larger holes were filled with Zn to prevent them being completely filled.

The advantages of this process are accurate sizes and shapes, reduction in annealing treatment due to low surface roughness, high strength and stiffness of BMG with the homogeneous and isotropic nature of the amorphous structure, strong anchoring of the BMG shell to the Si substrate due to the difference in the thermal expansion coefficients of the two materials, and higher Q factors of BMG. A disadvantage is surface contamination due to accumulated impurities on the surface, since the blow-molded 3D microshells are carried out in air [81].

**Figure 15:**
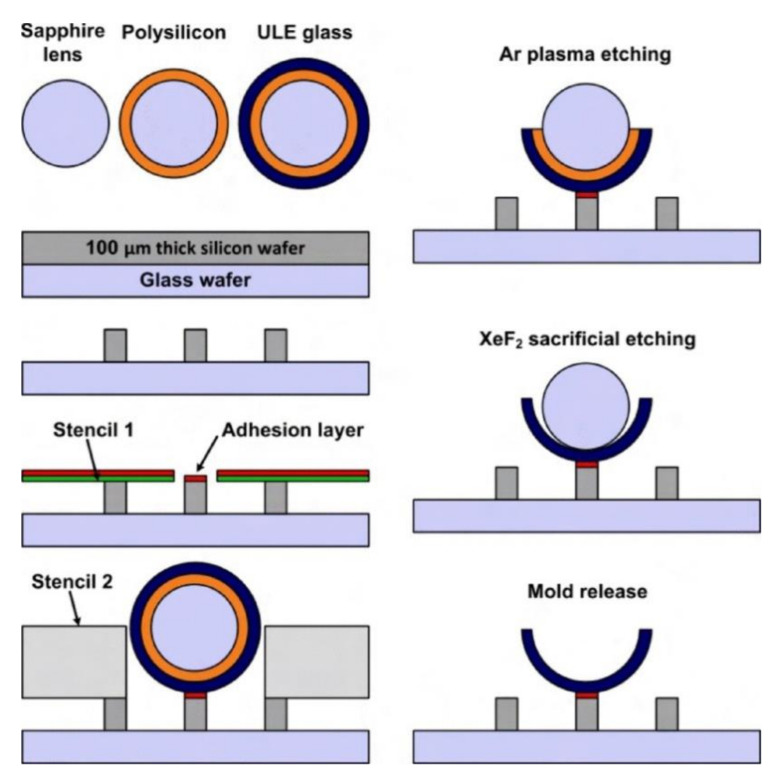
Detailed fabrication procedure of precision-curved micro-spherical shells (Xie et al. [54]) © 2022 IEEE. Reprinted, with permission from IEEE from Y. Xie, H. C. Hsieh, P. Pai, H. Kim, M. Tabib-Azar and C. H. Mastrangelo, “Precision-curved micro-hemispherical resonator shells fabricated by poached-egg micro-molding”, in SENSORS, 2012 IEEE, Taipei, Taiwan, 28–31 October 2012.

Xie et al. [54] and Rahman et al. [125] presented a micromolding batch fabrication technique to build HRGs via poached-egg micromolding (PEM). A layer of sacrificial sputtered silicon with a layer of sputtered ultralow expansion (ULE) glass (titania silicate glass) was used to coat the sapphire. The substrate was immobilized and patterned by lithography. DRIE was used to form the mold-bonding posts. The substrate was coated by a layer of sputtered silicon oxide. The coated lens was bonded on the post using a thin layer of bonding agent followed by bonding to the coated mold. The top half of the shell was ion-milled in Ar. Then, XeF_2_ etching was used to remove the sacrificial silicon layer. The hemisphere shell, with an attached substrate, was released from the molds by flipping the substrate [54]. Figure 15 shows the fabrication procedures for the resonator.

Xie et al. [54] experimentally measured the resonance frequency as 17,320 Hz with a Q factor of approximately 20,000. The stated advantage of this HRG was its high geometric accuracy and extremely uniform thickness.

Pai et al. [70,126] used silicate nitride to fabricate an HRG with a similar technique to Xie et al. [54] and Rahman et al. [125]. First, a low-stress LPCVD silicon nitride layer with etched circular openings was used to passivate a silicon wafer. The Si substrate was etched, and, after the creation of the hemispherical mold, the nitride mask was removed. The wafer was subjected to thermal oxidation to stop etching against XeF_2_ during sacrificial etching. The Al electrodes were patterned around the perimeter of the etched mold followed by sputter deposition of sacrificial amorphous silicon. Openings for pedestals/anchors were etched into the center of the mold. Sputtering SiO_2_ was deposited around the circumference of the mold, followed by patterning and etching. The sacrificial layer was etched by XeF_2_, resulting in free-standing SiO_2_ hemispherical shells on pedestals [70].

Gray et al. [80] used the conformal mold-coating technique with atomic-layer deposition (ALD) as the sole device material. Circular openings were patterned on a Cr/Au mask over a silicon substrate. The hemispherical molds were etched in the substrate, and the wafer was coated with ALD Al_2_O_3_. A thick photoresist was spun on and etched back, planarizing the molds. After etching the Al_2_O_3_ with buffered HF, only the Al_2_O_3_ protected by the photoresist remained. Finally, the silicon was etched back in an SF_6_ plasma, leaving the ALD shell supported by a silicon stem. The advantages of this process are low surface roughness, high symmetry of the shell with low radial deviations, a high Q-factor by reducing TED and anchor loss, uniform thickness control, very low surface roughness due to the use of ALD, minimum asymmetry due to the films’ high conformality, and long resonance decay times due to the ultrathin thickness. A disadvantage is the increased frequency, *f*_0_, due to a relatively high residual stress [80].

Shao et al. [127] used silicon dioxide with ALD coating. Shao et al. [128,129] and Sorenson et al. [130] used polysilicon, while Mehanathan et al. [131] used Invar (a nickel–iron alloy) to build HRGs with material deposited in a pre-etched cavity. Fabrication started by patterning a PECVD oxide layer to form the isotropic etch mask [130]. Isotropic etching of silicon created a hemispherical shell. An LPCVD low-stress silicon nitride layer was patterned to mask the top side and back side during KOH etching. In this process, the electrodes and hemispherical shell were self-aligned [128]. The advantages of this process are a high quality factor, highly symmetric structure, strong rotation rate sensitivity, and high drive amplitude due to the extremely small stiffness of the structure and thus large capacitive gaps. Disadvantages include the lack of a functional electrode system with controllable capacitive gaps [132], potential structural asymmetry, roughness of the surface and the complexity of the fabrication process [94,132]. The quality factor of the wineglass mode was measured as 8500 at 6.7 kHz with an as-built frequency mismatch of 105 Hz, with a scale factor of 4.4 mV/°/s.

Kanik et al. [132,133] used Pt57.5Cu14.7Ni5.3P22.5 BMG (Pt-BMG) to produce a wineglass HRG using a blow-molding technique. A hemispherical cavity was made in the borofloat glass mold with a hole at the center for the support stem. A conventional photolithography technique was used to fabricate the electrode structures. The hole was filled with a stainless-steel pin. Using a heated fixture, the top surface of the mold was sealed with a Pt-BMG blank, and the whole setup was heated, followed by the application of 0.69 MPa of ultrahigh purity N_2_ gas on the top surface of the blank. Soft-heated Pt-BMG was pressed into the mold by hydrostatic pressure. Cooled Pt-BMG was lapped and planarized to remove excess parts. The shell, with its steel stem, was separated from the mold by thermal expansion mismatch between the glass mold, M_2_ stainless-steel stem and the Pt-BMG shell. The advantages of this process are low surface roughness, high symmetry, uniform thickness, the low cost of fabrication, and elimination of energy dissipation mechanisms. Disadvantages include increased frequency mismatch due to strong coupling of the hemisphere to the stem and decreased performance due to residual stresses.

Bernstien et al. [134,135] used polycrystalline diamond to fabricate hemispheres by hot-filament chemical vapor deposition (HF CVD) in spherical cavities that were wet-etched into a high-temperature glass substrate. Hemispherical resonators showed a Q factor of up to 143,000 in wineglass mode with a frequency mismatch of 2 Hz, which was reduced to 0.3 Hz after laser trimming. Fabrication started by etching round cavities in the substrate glass, and the wet cavity etch was masked using a bilayer of polysilicon and UNCD (ultra-nano-crystalline diamond). The diamond and polysilicon films were stripped, and the diamond layer was electrically contacted at the anchor. A thick layer of low-pressure chemical vapor deposition (LPCVD) sacrificial polysilicon was deposited and patterned. Hot filament or plasma was used to deposit a diamond layer. This layer was masked with a thick layer of Cr, which was later etched and stripped. On the diamond electrodes, Cr/Au bond pads were deposited by evaporation and removed. The wafer was cut, and chips were released by timed etching. The advantages of this process are lack of crystal orientation due to use of glass substrate, a high Q-factor, since TED is reduced by using diamond (low coefficient of thermal expansion and high thermal conductivity), and reduction in anchor damping (the anchor is isolated by a slot cut into the base of the diamond hemisphere). Disadvantages include the existence of striations and bubbles in the glass and roughness of the sidewalls of the hemisphere, where most of the strain energy is located. Liu et al. [136] used a similar method and materials to fabricate an HRG.

Heidari et al. [57,137], Chan et al. [138] and Fonda et al. [139] used a combination of microelectron-discharge machining (EDM) and silicon micromachining techniques to fabricate HRGs with Si_3_N_4_ anchors, using microcrystalline diamond (MCD) as the device material because of its high stiffness and low TED characteristics. Piezoelectric and electrostatic excitation and optical detection were used as transduction methods. Fabrication started with deposition of a low resistivity silicon substrate with a metal hard mask. EDM was used to form a hemispherical silicon mold. The top surface of the silicon substrate was protected by a metal hard mask, and the inside surface of the hemisphere mold was etched. The etch-mask was stripped, and a layer of sacrificial SiO_2_ was deposited on the silicon mold. Etching the exposed diamond on the wafer surface left diamond within the hemisphere molds. The lithographically defined holes on the back of the wafer were opened. These openings were refilled with LPCVD, forming the anchors of the wineglass, and the silicon mold was removed. Advantages include excellent strength-to-density ratio, low surface losses, very low TED of diamond, and a low value of frequency mismatch. Disadvantages include potential structural asymmetry, roughness of the surface and complexity of the fabrication process [94,123].

Visvanathan et al. [45] and Li et al. [140] used the 3D-SOULE process (3D-capable and self-aligned process, combining batch-mode micro-ultrasonic machining, lapping, and micro-electrodischarge machining) to fabricate glass spheres in concave and mushroom shapes. The cavities in the steel substrate were micromachined using μEDM (micro-electro-discharge machining) and filled by epoxy. Stainless steel spheres were pressed into the epoxy. After curing of the epoxy, a silicon carrier substrate was bonded to the glass plate, and the glass spheres were bonded to the silicon substrate. A *μ*USM (micro-ultrasonic machining) process on the lapped glass spheres was performed to produce sharp edges along the rim of the fabricated structure. The sharp edges of the machined glass spheres were able to be removed by an additional lapping step, and the glass spheres were released from the silicon substrate. The advantage of the method includes utilizing advanced machining but the value of the reported Q is low.

Bhat et al. [141] and Torunbalci et al. [142] used the DICE (deep isotropic chemical etching) process for the fabrication of highly symmetric 3D hemispherical shell resonators (HSR) based on etching of a <111> silicon using a pop-up ring mask. The process started with molding a <111> silicon wafer [76]. A pop-up ring mask was formed on a <111> silicon substrate by etching LPCVD silicon nitride. Next, a hemisphere mold was formed by HNA-etching of silicon using the pop-up ring mask. After patterning a thermally grown structural layer of SiO_2_, the HSRs were released by XeF_2_. An advantage of this method of fabrication is a highly symmetric HRG.

Wan et al. [143,144] and Zhuang et al. [145,146] used polysilicon to fabricate a micro- HRG using the same method as Bernstein et al. [134,135]. The advantages of this method include elimination of the extra assembly process due to simultaneous fabrication of the shell and resonator, a simple fabrication method, and a large transduction area with a small and uniform gap size due to anchorage of the shell at the center with uniform circumferential electrodes. The performance of the resonator as a gyro in force-rebalance mode showed a frequency mismatch of 0.51 Hz, and a bias stability of 21.8 deg./h [144], while Zhuang et al. [145] reported performance of an HRG in open-loop mode with a resonance frequency of 28 kHz, a frequency mismatch of 0.009%, and a bias stability of 80 deg./h.

Luo et al. [147] and Ruan et al. [148] fabricated HRG using a chemical foaming process (CFP) in which the pressure of a foaming agent drives the viscous glass into the wineglass form. Ruan et al. [149] used a mode-matching method to eliminate angle bias error caused by stiffness asymmetry of the manufactured HRG; the experimental results showed a drift angle of 2.322 deg./h [148].

Zhang et al. [150] used a thermoforming process to fabricate an HRG from Pyrex. The process started by etching a concentric ring in a silicon wafer by ICP DRIE and then bonding Pyrex to this wafer. Then, the wafers were vacuumed, heated, formed and cooled.

## 4. Results and Discussion

The various geometric parameters, fabrication processes, materials and device performance characteristics discussed above are summarized in Table 1. The table depicts the most important geometrical and physical characteristics of the hemispherical shells reviewed. In total, 56 parameters of HRS, including fabrication method, device material, the rim diameter, surface roughness, thickness of the shell, working frequency at *n* = 2 mode, transduction method, and gyroscope response, including frequency split and q-factor, are tabulated. Further geometrical parameters include the height of the shell, the capacitance gap, number of electrodes, alignment of the shell and electrode, percentage of asymmetry of the shell or electrode arrangement, and supporting stem dimensions, as well as other physical parameters, such as the material used for the electrode, drift angle, scale factor nonlinearity and bias instability, phase error, the sensitivity of shell performance to the environment, and higher modes of vibration. However, as the scope of this review focuses on the device dimensions, materials, fabrication and resonator characteristics, only these parameters were selected to assist researchers in selection of the most suitable methods and materials.

As can be seen in Table 1, the majority of the HRG’s were fabricated using either a glassblowing or blowtorching method. Thus, it can be concluded that most of the methods were based on heat-forming. Among the papers analyzed, 91% of the material used for fabrication of HRG’s was glass in the form of fused silica 44%, polysilicon 14%, SiO_2_ 7%, Pyrex 5%, borosilicate glass 2%, bulk metallic glasses (BMG) 5%, Titania silicate glass 5%, glass 4%, with 11% built from diamond and less than 2% from Invar 36 and Al_2_O_3_. A total of 20% of the FS-based HRG’s were built by glassblowing and 80% using a blowtorching method. Thus, fused silica, in combination with glassblowing and blowtorching, were the most-often chosen material and methods used by researchers.

One of the most challenging tasks in building micro-HRG’s is to reduce the diameter of the shell as much as possible. The range of the rim diameter of the fused silica shell varied from 3.5 mm to 12 mm. The shells made from polysilicon had diameters of 0.7 mm to 1.4 mm, and the smallest diameter was built by a micromachining method. The smallest shell had a diameter of 0.1 mm, which was built from Al_2_O_3_ using a micromachining method, followed by 0.5 mm built from bulk metallic glasses using a blowtorching method. Although fused silica and thermoforming were the most used material and method used to build HRGs, respectively, the smallest diameter of the HRG built so far was 0.1 mm which was built using a micromachining method from Al_2_O_3_.

As previously mentioned, the symmetry of a shell is crucial for its performance. The geometry and designs pursued were inherently symmetrical. Some of the processes involved minimal masking and, therefore, minimal alignment error; some were self-aligning between the stem and shell, thus eliminating alignment errors. Therefore, one factor which cause asymmetry after fabrication was roughness of the surface. The less rough the surface, the more symmetry is obtained. Some showed automatically smooth shell surfaces. The roughness of the surface-built shells was not always quoted; however, based on available data presented in Table 1, it can be seen that the smoothest surface had an average surface roughness of 0.18 nm, belonging to the HRG built from fused silica using a blowtorching technique. The HRGs built from diamond had a surface roughness of 3 to 4 nm when built by a micromachining method. The smoothest HRG built by a glassblowing method had an average roughness of 0.217 nm. The HRG with the roughest surface was built from bulk metallic glass using a blowtorching method, with an average roughness of 43 nm. Therefore, overall, it can be concluded that blowtorching or glassblowing at the smallest scale improves the roughness and produces a smooth surface.

Reducing the thickness of the shell led to reduction in mass and consequent increase in the resonant frequency. The thinnest shell, having a thickness of 0.05 μm, belonged to the HRG built from Al_2_O_3_ using a micromachining method, followed by a diamond HRG with a thickness of 0.8 μm, also built using a micromachining method. The thinnest shell built from fused silica had a thickness of 20 μm, which was fabricated using a blowtorching method. The thinnest shell built by a blowtorching method had a thickness of 7 μm, which was built from bulk metallic glasses; that built by glassblowing having a thickness of 50 μm was made from borosilicate. Thus, it can be concluded that micromachining yields the thinnest shell followed by the blowtorching method.

The resonance frequency of the *n* = 2 mode of vibration is an important performance parameter of Coriolis vibratory resonators. The maximum resonance frequency of 412 kHz corresponded to the HRG built from polysilicon using a micromachining method. The maximum resonance frequency of the fused silica HRG was 105 kHz, built using a glassblowing technique. The maximum resonance frequency achieved by the blowtorching method was 18.7 kHz, using a fused silica method. Thus, it can be concluded that micromachining yields the maximum resonance frequency followed by the glassblowing method.

Frequency mismatch is a negative performance parameter of Coriolis vibratory resonators, especially for mode match operation, and should be as low as possible. The minimum absolute value of frequency mismatch achieved by a glassblowing technique was 0.15 Hz (0.0005% of the resonance frequency of the HRG) using borosilicate. The minimum frequency mismatch achieved by a micromachining method was 0.51 Hz which was 0.004% of the resonance frequency of the HRG built from polysilicon. The minimum frequency mismatch of the fused silica HRG is 0.9 Hz was 0.007% of the resonance frequency of the HRG built by a blowtorching method. Thus, it can be concluded that glassblowing techniques yield the minimum frequency mismatch, followed by micromachining methods.

Achieving the highest quality factor is of paramount importance for researchers. The maximum quality factor was 5.2 × 10^6^ corresponding to the HRG built from fused silica using a blowtorching method. The highest quality factor achieved by a glassblowing technique was 1.14 × 10^6^ of the HRG built by fused silica, and the maximum quality factor achieved by the micromachining method was 412,000, corresponding to an HRG built by polysilicon.

## 5. Conclusions and Future Outlook

In this study, all recent research studies on different materials and fabrication methods used for the fabrication of hemispherical resonator gyroscopes (HRGs) were reviewed in full detail. The basic theory of HRGs was discussed, followed by consideration of the incident that triggered the initiation of glassblowing techniques. The integration of electrodes with glassblowing techniques, the use of fused silica, and other material fabrication methods were also examined. The inherent advantages and disadvantages of different HRGs were outlined to develop an understanding of the appropriate selection of the best possible HRGs within the limitations of the available materials and fabrication methods. Key issues identified for the design and development of HRG resonators were the attainable resonant frequency, the quality factor, and the frequency split. A summary of the papers reviewed, citing the fabrication methods, materials and geometrical parameters with HRG dynamic characteristics, which can be used as guidance to help select the best material and fabrication method based on the desired operational frequency and expected Q-factor, was presented. Based on this summary, it can be concluded that fused silica, in combination with blowtorching, are the most often selected material and method, respectively, used by researchers. The micromachining method yields the smallest and thinnest HRGs with maximum resonance frequency, so far blowtorching yields hemispherical resonator with highest quality factor and glassblowing technic yields the hemispherical resonator with lowest frequency mismatch. HRGs built from fused silica have the minimum surface roughness. The next point that should be addressed is packaging. Based on the studies reviewed, HRGs should be able to be applied in daily life. Therefore, they must be packaged in such a way that they can be easily stored, handled, and replaced. Although there have been many significant developments in recent years, there are still many obstacles to overcome to achieve micro-HRGs for wider adoption, mainly due to fabrication and implementation complexities. Although wafer-scale glassblowing has achieved a critical milestone, wafer-scale co-fabrication of electrodes and vacuum packaging remains a challenge. Finally, the price of fabrication should be in a reasonable range so that the final product will be affordable for industry.

## Figures and Tables

**Table 1:** Comparison of different HRG device characteristics.

Fabrication Method	Material	Diameter (mm)	Surface Smoothness (nm)	Thickness (μm)	Resonance Frequency *n* = 2 (kHz)	Transduction Method	Resonator Response		Ref.
							Frequency Split, *n* = 2 Hz (Percentage of Resonance Frequency)	Q-Factor	
Micro- glassblowing	Borosilicate glass	4.4	Sa = 0.23	50	27.4	Integrated radial cylindrical electrode	0.15 (0.0005%)		Senkel et al. [86,90]
	Fused silica	7		500	105	Electrostatic, out-of-plane electrodes	14 (0.01%)	1.14 × 10^6^ (<20 mTorr)	Senkal et al. [75,83,92]
									Senkal et al. [93]
		7.8		86.2	9.6	Electrostatic actuation and detection	70 (0.73%)		Asadian and Shkel [96]
					9.68	Electrostatic actuation, capacitive detection	8.2 (0.08%)	~219,000	Asadian et al. [47]
		12		200	4.3		~20 (0.47%)	~1.0 × 10^6^	Shi et al. [117,118]
	Pyrex	1			1660	Integrated electrodes		2700 (0.4 mTorr)	Giner et al. [95]
Micrometer-blow-torch-molding	Fused silica	5		t_rim_ = 70	10.45	Assembled discrete electrode	14 (0.13%)	249,000 (<1 mTorr)	Cho et al. [69]
			R_q_ = 0.53	90–100	8.2~18.7		19.6~839.63 (0.2~4.5%)	294,450 (<1 mTorr)	Cho et al. [98]
			<1.0	t_rim_ = 60	8.8		6.7 (0.08%)	1.2 × 10^6^ (<1 mTorr)	Cho et al. [82]
				100 t_rim_ = 70	10.47	Assembled discrete electrode	14 (0.13%)	244,750 (<1 mTorr)	Cho et al. [72]
				t_rim_ = 80	16.66	Radial spherical electrodes	10 (0.06%)	224,930	Cho et al. [74]
			R_q_ = 0.53	100	8.21		20 (0.24%)	213,604 (<1 mTorr)	Cho et al. [99]
				88.4–169 t_rim_ = 90	22.57		137 (0.61%)	2.55 × 10^6^ (<1 mTorr)	Cho and Najafi [109]
				100					Shiari et al. [101]
			0.24		12.9	Flat electrode		271,669 (<100 mTorr)	Nagourney et al. [102]
			0.18	t = 30–100	9.78			3.1 × 10^6^	Nagourney et al. [103]
					10.46		10 (0.10%)	72,870 (<1 mTorr)	Woo et al. [104]
		10		200	5.67		3.29 (0.06%)	5.2 × 10^6^	Cho et al. [50]
					5.80	Out-of-plane drive/sense transduction	2.1 (0.04%)	1.27 × 10^6^ (<1 mTorr)	Singh et al. [52]
Blow-molding	Bulk metallic glasses (BMG)	0.50	<2	7~15					Sarac et al. [81]
Poached-egg micromolding	Titania silicate glass (ULE Glass)	1		1.2	17.32			20,000 (100 mTorr)	Xie et al. [54]
					5.84	Integrated electrostatic electrodes		730 (Atmosphere)	Rahman et al. [125]
Micromachining	SiO_2_	0.5	<5		21.9	Electrostatic driving/sensing	5 (0.02%)	22,000 (50 mTorr)	Pai et al. [70]
				1	22			>10,000 (50 mTorr)	Pai et al. [126]
Conformal mold coating	Aluminum oxide (Al_2_O_3_)	0.10		0.05	60		100 (0.17%)	1000–2000 (0.01 mTorr)	Gray et al. [80]
Deposition of thin film in mold	SiO_2_	0.74		2	113.9	Self-aligned capacitive electrodes		5600 (In vacuum)	Shao et al. [127]
	Polysilicon	1.2		0.7	6.7		100 (1.5%)	8500 (In vacuum)	Shao et al. [128,129]
				0.66	412			8000 (In vacuum)	Sorenson et al. [130]
	Invar-36	0.78		5.2	29.08		27 (0.52%)	7567 (In vacuum)	Mehanathan et al. [131]
Blow-molding technique	Pt-BMG	3	R_a_ = 43	t_rim_ = 30	9.4	Electrostatic, self-aligned electrode and capacitive readout	8 (0.09%)	7800	Kanik et al. [132]
			R_a_ < 1	70	13.94		5 (0.04%)	6200	Kanik et al. [133]
Deposition in mold	Polycrystalline diamond	0.7			22.45	Actuated electrostatically	1038 (4.6%)	11,500 (0.25 mTorr)	Bernstien et al. [135]
		1.4			16		0.3 (0.002%)	143,000 (<0.01 mTorr)	Bernstien et al. [134]
			R_a_ < 3		81.3	Electrostatic excitation and capacitive detection	699.18 (0.86%)	402 (in air)	Liu et al. [136]
Micro-electro discharge Machining and silicon micromachining techniques	Polycrystalline diamond	1		0.8–1.0	18.32	Electrostatic excitation and optical sensing	5 (0.03%)		Heidari et al. [57,137]
			4						Chan et al. [138]
		1							Fonda et al. [139]
3D-SOULE process (combining batch mode micro-ultrasonic machining, lapping and micro- electro-discharge machining)	glass	1.0			1379			345 (in air)	Visvanathan et al. [45]
		1.0			1379				Li et al. [140]
Deposition in a deep isotropic chemical-etching mold	SiO_2_	1.2		1.1				206 (In air)	Bhat et al. [141]
		1.8			100.65		512 (0.51%)	31,542 (0.025 mTorr)	Torunbalci et al. [142]
Deposition in a pre-etched low-resistivity p-type single-crystal crystal <111> silicon mold	Polysilicon	1.3		1.43	14.18	Spherical electrodes	0.51 (0.004%)	22,000 (0.03 mTorr)	Wan et al. [143]
								15,200	Wan et al. [144]
		0.78		1.8	28	Integrated 3-D curved electrodes	2.6 (0.009%)	14,365 (6.75 mTorr)	Zhuang et al. [145]
		1.3		1.5	13.65		4 (2.93%)	22,000 (0.075 mTorr)	
		0.79		1.50		Self-aligned spherical capacitive electrodes			Zhuang et al. [146]
Micro-blow-torching process	Fused Silica	3.5		100	10.49	Out-of-plane electrodes	430 (4.1%)	12,558 (In vacuum)	Li et al. [110]
Micro-blow-torching process and femtosecond ablation		7.0		t_rim_ = 100	12.55		0.9 (0.007%)		Lu et al. [111]
Micro-blow-torching process with whirling platform and femtosecond ablation					6.91		12.1 (0.18%)	36,940 (In vacuum)	Lu et al. [112]
		6.7		t_rim_ = 100			11.3 (-)		Lu et al. [113]
		7.6		t_rim_ = 20	10.69		45.55 (0.43%)		Li et al. [114]
		7.9	R_a_ = 0.404	t_rim_ = 100	6.91		12.1 (0.18%)	36,940 (at vacuum)	Li et al. [115] and Xiao et al. [94]
		12		200	4.34		17.8 (0.41%)	1.18 × 10^6^	Shi et al. [118,119]
Glassblowing	Pyrex 7740	2.0	Ra = 0.217 R_q_ = 0.296	60.68		Annular electrodes			Wang et al. [123]
		1.0~1.2	0.26 ± 0.06	67.5~76.6	1.26~1.58	Eaved-shape electrode			Wang et al. [124]
Chemical foaming process		7	Ra = 0.332 R_q_ = 0.421	50					Luo et al. [147]
